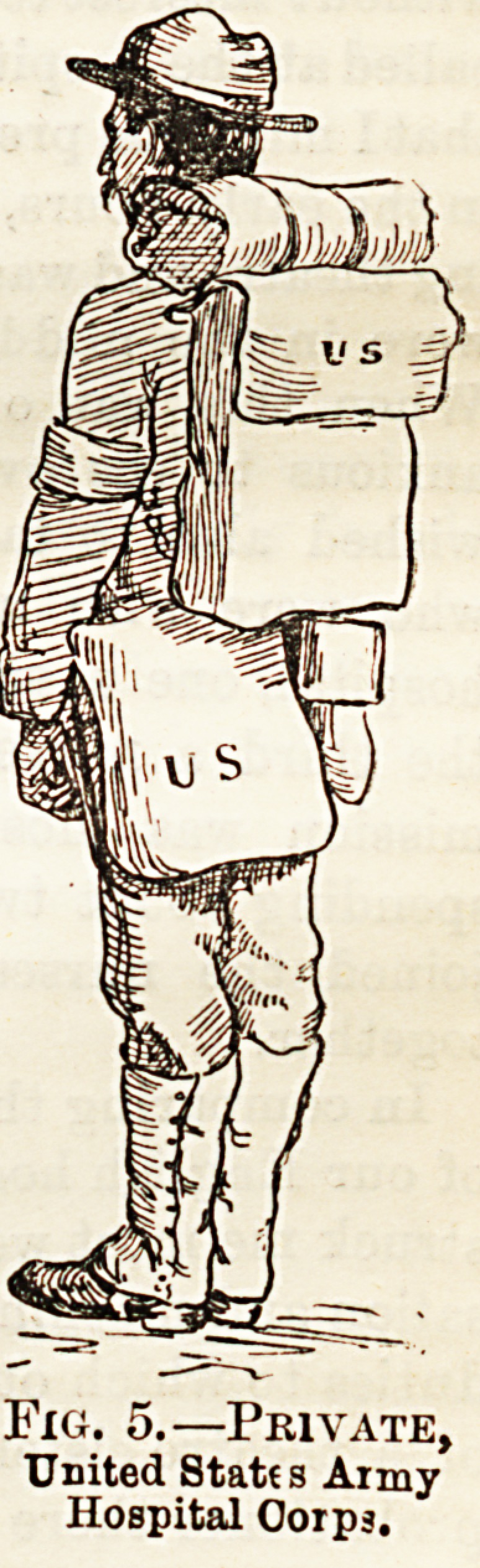# Medical Aid in Time of War

**Published:** 1898-09-24

**Authors:** 


					448 THE HOSPITAL, Sept. 24, 1898.
The Institutional Workshop.
MEDICAL AID IN TIME OF WAR.
AMBULANCE SERVICE IN THE UNITED STATES
- ARMY.
Very elaborate and effectual arrangements are made
by the United States Army Medical Department for the
cara of their sick and woanded. The drawings here re"
produced are not without interest as showing what attempts
are made to render medical aid promptly and efficiently on
the field of battle in modern days.
* The first illustration shows the folding furniture used in
the field hospitals and in the medioal officers' quarters. The
field hospital consists of a wall tent and a hospital tent, the
medical officer having his quarters in the former, while in
the latter are kept the hospital records and dispensary
supplies. Here also patients are seen. The ward of the
field hospital consists of two hospital tents, pitched end to
end, and contains twelve cots, other furniture being limited
to chairs and tables, such as those depicted in the drawing.
A hospital tent called the squad tent is used for the detach-
ment of the Hospital Corps on duty, usually eight men in all.
The second drawing shows the "travois," with litter in
place, the litters beiDg interchangeable with those used on
the field and in the ambulances for the more convenient
transportation of the wounded.
Illustration No. 3 shows the pack-saddle in use, with
medical and surgical chests in position for transport.
The army oven here pictured is known as the " Buzzicot.""
Cooking for the field hospital is done in a wall tent and in
the open air in front. When packed the oven answers the
purpose of a box, in which the smaller oooking utensils can
be packed. When over the fire one oven may be covered by
the other, and should it be necessary or desirable to bake the'
contents on top?as, for instance, in the case of bread?live>
coals may be placed on the top oven, and this in turn serves
the purpose of a convenient place for warming or cooking
additional articles of diet.
The last illustration shows the field equipment of a private-
in the United States Army Hospital Corps. The following
lists give the articles contained in the hospital corps and
orderly pouches, the former being carried by every member
of the corps, whilst the latter are carried by the medical
officer's orderly only :?
Pouch?Hospital Cobps.?1 cz. of aromatic spirits of
ammonia; 4 roller bandages; 1 candle in tin box ; 1 first
aid packet; 1 dressing forceps*; 1 iodoform sprinkler; 1
jack-knife ; 2 ozj. sublimated lint; 1 paper medium needles* ;
^ cz. carbolizad petrolatum; 1 paper common pins*; 6 safety
pins*; 1 spool of adhesive plaister; 1 medium pair scis-
sors* ; 2 wire splints; 2 small sponges in bag; 20 yarda
linen thread; 2 field tourniquets; 2 tza. boracio wool.?
*Articles marked thus are contained in special case.
Pouch?Obderly.?1 cz. of aromatic spirits of ammonia;
2 roller bandages; 1 pus basin ; 1 medicine case, with tablets;
1 elastic catheter (No. 8) ; 4 czs. chloroform; 1 first aid
packet; 1 medicine glass; 2 cz?. sublimated lint; & cz.
carbolized petrolatum; 1 paper common pins ; G safety pins ;
1 medium pair scisaors; 2 small sponges in bag ; 1 hypoder-
mic Byringe, with tablets; 1 book of diagnosis tags, with
pencil; 1 Esmarch tourniquet; 2 ozs. boraoic wool.
AMERICAN RED CROSS SOCIETY.
The hospital records of the lato war will be interesting
reading when they come to be published. The resources of
the Army and Navy Medical Departments must haye been
tried to the uttermost with the numbers of sick and
wounded under treatment. The Amerioan Red Cross
Society has clearly done excellent supplementary work,
and the aid rendered by Miss Barton and her helpers
has met with ample recognition on the part of the army
medical authorities. A statement has been published by
MEDICAL AID IN TIME OF WAR. A hosPitaI tent called the squad tent is uaed for the detach-
ment of the Hospital Corps on duty, usually eight men in all.
AMBULANCE SERVICE IN THE UNITED STATES The second drawing shows the "travois," with litter in
ARMY. place, the litters being interchangeable with those used on
Very elaborate and effectual arrangements are made the field and in the ambulances for the more convenient
by the United States Army Medical Department for the transportation of the wounded.
cara of their sick and wounded. The drawings here re* Illustration No. 3 shows the pack-saddle in use, with
medical and surgical chests in position for transport.
The army oven here pictured is known as the " Buzzicot.'*
Cooking for the field hospital is done in a wall tent and in
the open air in front. When packed the oven answers the
purpose of a box, in which the smaller cooking utensils can
be packed. When over the fire one oven may be covered by
the other, and should it be neoessary or desirable to bake th&
contents on top?as, for instance, in the case of bread?live>
coals may be placed on the top oven, and this in turn serves
the purpose of a convenient place for warming or cooking
additional articles of diet.
The last illustration shows the field equipment of a private*
in the United States Army Hospital Corps. The following
lists give the articles contained in the hospital corps and
orderly pouches, the former being carried by every member
of the corps, whilst the latter are carried by the medical
officer's orderly only :?
Pouch?Hospital Corps.?1 ez. of aromatic spirits of
? ammonia; 4 roller bandages; 1 candle in tin box; 1 first
Fig. 1. Field Hospital Furniture. aid packet; 1 dressing forceps*; 1 iodoform sprinkler; 1
. jack-knife ; 2 ozj. sublimated lint; 1 paper medium needles*
produced are not without interest as showing what attempts ^ cz carbolized petrolatum; 1 paper common pins* ; 6 safety
are made to render medical aid promptly and efficiently on pins*; 1 spool of adhesive plaister; 1 medium pair scis-
the field of battle in modern days. sors* ; 2 wire splints; 2 small sponges in bag; 20 yards
* The first illustration shows the folding furniture used in linen thread; 2 field tourniquets; 2 tza. boracio wool,
the field hospitals and in the medical officers' quarters. The "Articles marked thus are contained in special case.
field hospital consists of a wall tent and a hospital tent, the Pouch Orderly. I t z. of aromatic spirits of a mmonia;
2 roller bandageB; 1 pus basin ; 1 medicine case, with tablets;
1 elastic catheter (No. 8) ; 4 tzs. chloroform; 1 first aid
packet; 1 medicine glass; 2 cz3. sublimated lint; & cz.
carbolized petrolatum ; 1 paper common pins ; 6 safety pins;
1 medium pair BoisBors; 2 small sponges in bag ; 1 hypoder-
mic syringe, with tablets; 1 book of diagnosis tags, with
pencil; 1 Esmarch tourniquet; 2 ozs. boraoic wool.
AMERICAN RED CROSS SOCIETY.
The hospital records of the lato war will be interesting
reading when they come to be published. The resources of
the Army and NaYy Medical Departments must have been
Fig. 2.?" Travois, " with Litter,
Sept. 24, 1898. THE HOSPITAL, 449
Surgeon-General Sternberg deprecating the newspaper re-
ports of hostility to the society on the part of the Army
Medical Department, and expressing strong appreciation of
the valuable services rendered by the Red Cross. He holds
the view that wcmen nurses are an encumbrance to troops
during active operations, but adds that when serious sickness
developed and it became necessary to treat typhoid fever
cases in the field hospitals, he gladly accepted the services
Fig. 3.?Pack Saddle.
of trained female nurses for the division field hospital, and
in the general hospitals they were employed from the first.
In a letter to a Philadelphia medical paper, Major Clarke,
of the Army Medical Service, writing from Camp Cuba
Libre, Florida, also speaks gratefully of the services of the
Red Cross Sooiety. " No cne," he says, " should write from
the front without giving great oredit to the Red Cross
Fig. 4.?''BrzzicoT " Oven.
Society for the great work it is doing. Without the aid^ of
this society it would hare been difficult to care for the Bick
in this hospital. Each day the Red Cross gives u3 fifty
gallons of milk, two thousand pounds of ice, thirty d(z;ns
of eggs. Besides this it has built for ub storehouses, floored
our tents, giTen U8 hundreds of nigbt-shirts, pyjamas,
pillows, sheets, buckets, bedpans, and dczans of other articles
necessary for the care of the sick. In military channels the
stream of supplies flows slowly and with many stops and
much formality, but Dr. Kent, who represents the Red
Cross here, gives freely and without delay. He has no
regulation that prescribes how many bedpans one division
must use, but gives all that are needed. This week, with
the people of Jacksonville furnishing the motor power, he ia
going to put electric fans in all the tents."
Conspicuous companions to the fleets have been the hospital
ships, painted in conformity with the articles of the Geneva
Convention?white, with abroad green stripe?and flying at
the fore the Geneva cross, token and sign of a merciful
mission, to be respected alike by friend and foe. At the
first mention of war the United States Navy Department
obtained possession of a new steel vessel, designed by Mr.
Horace See for passenger traffic between New York
and New Orleans, and built by the Newport News
Shipbuilding and Dry Dock Company, changed her name
from the "Creole" to the more appropriate one of the
" Solace," and refitted her for hospital purposes. A descrip-
tion of the "Solace " in a recent Medical Record gives her
displacement as that of 3,800 tons, with a length of 375 feet,
and a speed power of from 14 to 17 knots. The holds and lower
decks are devoted to ship stores and provisions, and store-
rooms for medical and surgical supplies. Here also a large
steam steriliser finds a place. The crew find quarters on the
main deck forward, and abaft these is the chief ward, con-
taining ninety-two bunks, accessible on all sides, and further
afc again a large saloon with state-rooms on either side,
this being the accommodation intended for convalescent
officers. The main ward is well ventilated and lighted, and
supplied, in addition to numerous portholes and a large
hatch, with louvres and ducts connecting with blowers, en-
suring at all times a good renewal of fresh air. Hot and cold
water is laid on in abundant supply, and bath-rooms and
lavatories are in close proximity. Above the ward is the
operating-room, communicated with by a lift large enough
to carry a cot or wheeled Btretcher. On the main deck also
are the state rooms for most of the nurses attached to the
ship, and the assistant medical officers, besides cold-storage
rooms, ice houses and ice machines, the steam laundry, and
an emergency ward fitted with fifty
swinging cots.
The large and well lighted operating
room on the upper deck forward is,
as already mentioned, over the big main
ward. It is fitted with two operating
tables of the regulation pattern, and
with every appliance of modern surgery,
fifctiDgs and instruments being of the
newest and best make. The deok is
covered with interlocking rubber tiles,
which have the great advantage of
affording a firm foothold when the ship
is pitching, and are also capable of the
most thorough cleansing. The bulkheads
are painted with enamel paint to allow
of the required cleansing. Next to
the operating room comes the doctors'
sterilising room and the dispensary.
The latter is also the telephone centre,
communicating by this means with the
wards and sick rooms and the surgeons'
quarters. On this deck also are the
quarters of the senior surgeon and the
chief dispenser, the officers' mess room,
with a number of state rooms devoted to the use of injured
officers, and in the after deck-house a saloon for convalescent
V\N ^
Fig. 3.?Pack Saddle.
'< ? ar
pIG- 4 __<? Bczzicot " Oven.
Yj S
Fig. 5.?Private,
United States Army
Hospital Corps.
450 THE HOSPITAL. Sept. 24, 1898.
enlisted men, communicating with the emergency ward
below.
The promenade deck contains the quarters of the captain
and deck officers, and offices for the executive officer, the
senior medical officer, &o. Arrangements were made for
utilising the after part of the promenade deck as an isolation
ward, screened off by awnings, should any outbreak of in-
fectious disease show itself. Plentiful water provision was
made in fitting out the " Solace " as a hospital vessel. She
carries a large distilling plant, and a storage capacity for
27,000 gallons of fresh water. Of course, electric light is
laid on in every part of the ship, and she is also fitted
with search, mast head, and Bide lights, and supplied with
hand- steering gear for use in emergency. On the upper deck
she carries two steam launches, fitted with platform decks
for the easy conveyance of sick and wounded in cots or
hammocks. The launches are, like the " Solace" herself,
painted white and green.
Since the commencement of the war a second ship, the
" Relief," has been requisitioned and fitted up for hospital
work, and the addition of a third vessel has been under con-
templation, so that there can be no doubt as to the efficiency
of the care and attention given by the United States Navy
Department to the officers and men who have fought so well
for their country in the recent war, when once they have
reached the hospital ships.

				

## Figures and Tables

**Fig. 1. Fig. 2. f1:**
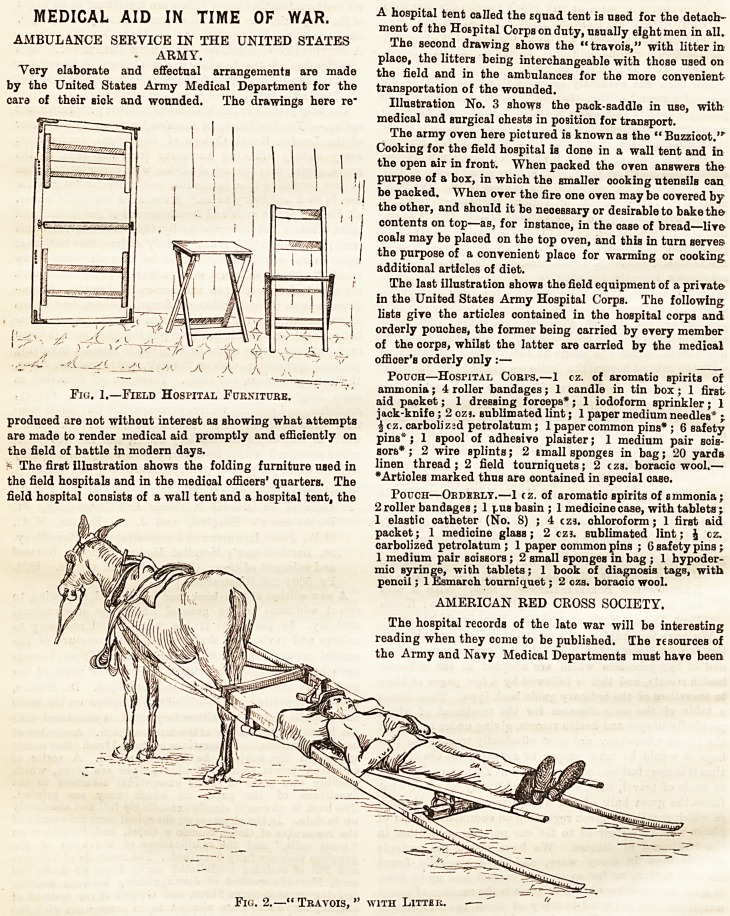


**Fig. 3. f2:**
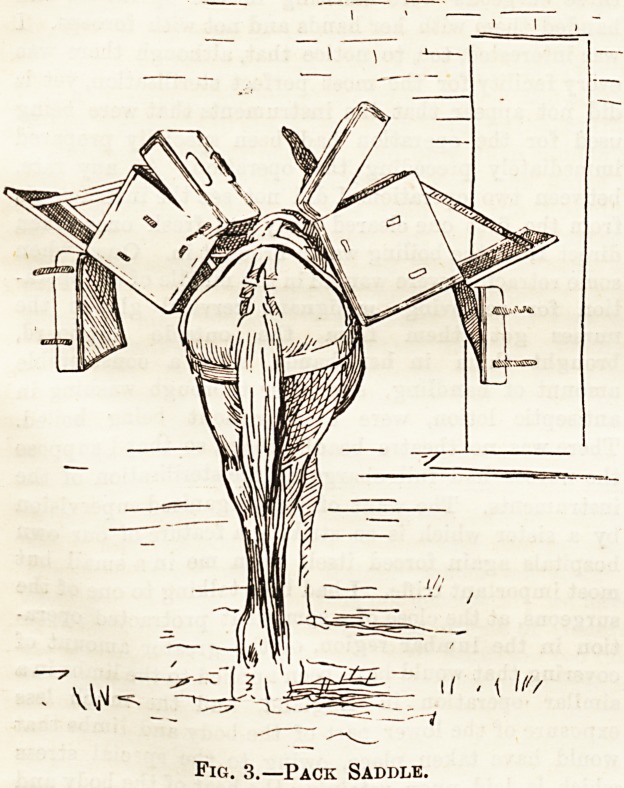


**Fig. 4. f3:**
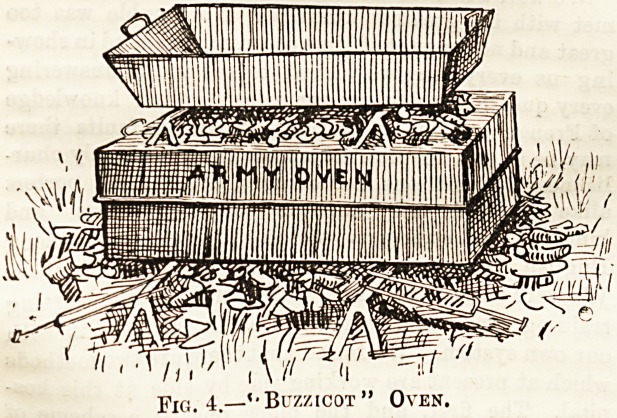


**Fig. 5. f4:**